# An Infected Dog Bite With Neisseria canis: A Case Report and Review of the Literature

**DOI:** 10.7759/cureus.52712

**Published:** 2024-01-22

**Authors:** Richard Giovane, Luis Pernia, Traci Guy, Hannah Blevins

**Affiliations:** 1 Family Medicine, University of Alabama, Tuscaloosa, USA; 2 Plastic Surgery, DCH Regional Medical Center, Tuscaloosa, USA; 3 Pharmacology, Northport Medical Center Pharmacy Department, Northport, USA; 4 Pharmacology, Samford University McWhorter School of Pharmacy, Birmingham, USA

**Keywords:** infectious cellulitis, drug allergy recording, site of infection, neisseria spp, dog bites

## Abstract

*Neisseria *is a common bacteria that colonizes in humans. Of the 11 species, only two, *N. meningitidis* and *N. gonorrhea, *are pathogenic. Although sparse, there are case reports of other Neisseria species causing infections in humans. *Neisseria canis*, which is a part of normal flora in the mouths of dogs and cats, has been shown to have potential to be pathogenic in humans. The standard treatment for dog and cat bites is oral amoxicillin/clavulanic acid (Augmentin) or IV ampicillin/sulbactam (Unasyn). However, in cases where the patient has multiple antibiotic allergies, careful antibiotic selection must be made to ensure resolution of infection.

## Introduction

*Neisseria* are gram-negative, catalase- and oxidase-positive diplococci bacteria that colonize the surfaces of animals [1]. The pathogenesis of *Neisseria* is initially from the adherence to epithelial cells through type IV pili. Once this is accomplished, *Neisseria* replicates and forms microcolonies. During this stage, *Neisseria* releases peptidoglycan, lipooligosaccharides and outer membrane vesicles as fragments [1,2]. More specifically, the lipooligosaccharide functions as an endotoxin and induces a response from the host immune system [2,3]. This in turn will release toll-like receptors that signal epithelial cells, macrophages and dendritic cells, which then activate cytokines and chemokines [1,3].

Eleven known species of Neisseria colonize humans; only two, *N. meningitidis* and *N. gonorrhea,* are pathogenic to humans [4]. There exist other species that are a part of normal flora in other animals. However it is rare for these species to be infectious to humans. Although *N. canis*, another species, is a part of normal flora for dogs and cats, it still demonstrates the potential for pathogenicity in humans through puncture wounds in rare circumstances [5]. 

## Case presentation

We report a novel case of an infected hand caused by a dog bite due to *Neisseria canis*. The patient is a 51-year-old female with a past medical history of hypothyroidism who presented to the emergency room after sustaining a dog bite from her daughter’s pet German Shepherd. The patient reported that previously she has had no issues with the German Shepherd, and reported an overall mild demeanor of the dog at baseline. When the bite occurred, she reports that she was sitting in the living room and the German Shepherd started attacking her randomly. The patient suffered from bite wounds to her arms bilaterally and her face. She immediately came to the emergency room to seek medical attention and was evaluated in the emergency room. The patient was evaluated at bedside and several superficial lacerations as well as scratch marks over the patient's face were noted bilaterally. Regarding the patient’s forearms, several deep lacerations were noted on her left forearm and hand, but she had full range of motion and no loss of sensation. On the patient's right forearm there was a deep bite mark as well as an open fracture at the trapezium at the base of the first metacarpal. On initial presentation, the patient's vital signs were stable; temperature of 98.3 F, pulse 102, respiratory of 16 and blood pressure 123/65. On physical exam, the patient had significant tenderness and pain on her right hand and forearm as well as numbness over the base of the right thumb as well as her dorsal right forearm. Her radial pulses were two plus bilaterally. Her cardiac exam revealed a regular rate and rhythm without murmurs. Her lungs were clear bilaterally. 

**Figure 1:**
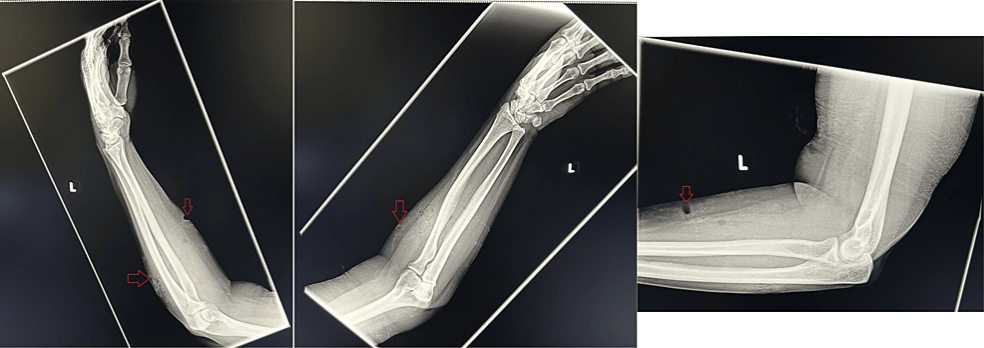
X-ray of right forearm

**Figure 2:**
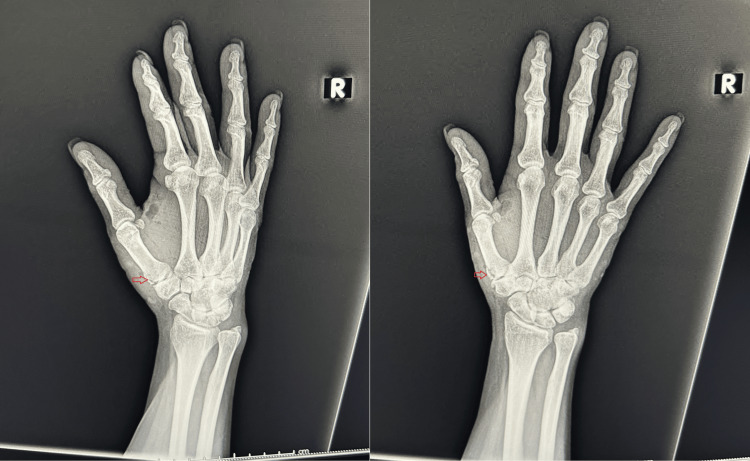
X-ray of right hand

The patient was sent for X-rays on her forearms. The X-rays showed significant soft tissue lacerations and subcutaneous emphysema over the right forearm (Figure 1) and a follow-up right-hand X-ray was taken which showed a non-displaced fracture involving the base of the first metacarpal and its articulation with the trapezium with associated tissue injury (Figure 2). An X-ray of the left hand and forearm was done as well, which were both unremarkable. 

While in the emergency room, the patient did receive a tetanus vaccine due to the patient not knowing her tetanus status. The patient also reported that the dog was up to date on all vaccines and lived mostly inside of the house. 

Lab work was done on the patient initially which showed a WBC of 7.72 10^3/uL, hemoglobin of 12.1 g/dL, hematocrit 35.8%, platelet count of 285 10^3uL. A comprehensive metabolic panel (CMP) was also done which showed sodium 138 mmol/L, potassium 3.4 mmol/L, creatinine of 56 mg/dL, glucose 92 mg/dL, aspartate aminotransferase (AST) 71 IU/L and alanine aminotransferase (ALT) of 37 IU/L.

Plastic surgery was consulted who recommended the patient go to the operating room immediately for debridement of wounds due to fear of septic arthritis. During surgery, wound cultures were taken. It was noted during surgery that the patient had a wound on the volar side of the thumb which lacerated the abductor pollicis brevis and opponens pollicis. Furthermore, the wound penetrated directly into the metacarpal trapezial joint with fracture fragments. This area was divided and flushed out using Irrisept solution. Regarding the patient’s left forearm and hand, it was noted to have the most severe wounds on the forearm. A ripping type lacerations of the flexor carpi radialis and the palmaris longus muscle with penetration into the tendon of the flexor carpi radialis were noted as well as injury to the brachioradialis and the extensor carpi radialis. These areas were flushed out with 450cc of Irrisept. Excisional debridement was done with a #15 scalpel. Hemostasis was achieved using bipolar cautery. Finally a sterile occlusive dressing was applied on the left forearm and on the right forearm as well as on the left hand. The left hand was then placed in a short arm static volar thumb spica splint with the wrist in dorsiflexion and the thumb in abduction.

Postoperatively, the patient was placed on IV doxycycline 100 mg twice a day per infectious disease recommendation. Ceftriaxone, metronidazole and vancomycin were added due to the patient having postoperative fever of 101.1 F within 24 hours as well as having more pain to her forearms bilaterally during post-operative day 1. Infectious disease did recommend stopping ceftriaxone, metronidazole and vancomycin and keeping the patient on monotherapy of doxycycline. On post-op day 2, the patient’s pain was well controlled and her pain was adequately controlled however the patient did have a fever of 100.4 F. On postoperative day 3 the patient had a fever of 100.9 F overnight. Wound cultures came back negative for methicillin-resistant Staphylococcus aureus (MRSA) but positive for *N. canis *and *Pasturella *sp. Further microbiology testing was requested for *N. canis* and *Pasturella *sp., but neither of the organisms thrived for susceptibility testing. Of note, blood cultures remained negative during hospitalization. On postoperative day 4, the patient continued to have a fever of 101 F. It was decided to order a CT scan of the face to rule out a facial abscess however it only showed multiple lacerations without any acute abnormality. On postoperative day 5, the patient continued to have fever of 102 F but her WBC was within normal limits. It was decided to escalate antibiotic therapy to ceftriaxone, doxycycline, and clindamycin for broad spectrum coverage as well as the patient still having fever. At this time, the patient did lose IV access and required a peripherally inserted central catheter (PICC) line. Also at this time, the patient developed oral thrush in which she was started with PO nystatin which relieved her symptoms. The patient was closely monitored and she continued to improve in regard to pain as well as bilateral upper extremity mobility. More importantly, once the patient was started on ceftriaxone, doxycycline and clindamycin, she remained afebrile. The patient was observed for 48 more hours and she was discharged home with PO doxycycline 100mg twice a day for four days, cefdinir 300mg twice a day for five days and clindamycin 300mg four times a day for seven more days (to complete a 10-day course of all three antibiotics).

Of note, the patient did have allergies to the following antibiotics: ciprofloxacin, penicillin, sulfa, and nitrofurantoin. Upon further questioning about these allergies, the patient reported anaphylaxis in the past with ciprofloxacin, penicillin, and sulfa. She also reported mouth sores with nitrofurantoin. 

*Pasteurella *sp. antibiotic sensitivity was attempted, but resulted in failure to thrive. *N canis* was considered as “normal flora,” therefore no antibiotic sensitivities or minimal inhibitory concentrations (MIC) were able to be conducted. 

A follow-up call with the patient was done on day 22. The patient reported that her wounds were healing well with no scabbing or seepage. She did however report decreased functionality with her right thumb and index finger as well as having pain in her right hand and left arm. She reported her strength in her right hand and left arm are reduced from her baseline; however, she has been able to return to work. Lastly, she reported that she is having some residual numbness in her right thumb, but can feel temperature extremes and most touches. She reported close follow-up with a local orthopedic surgeon to see if there are any further interventions indicated.

## Discussion

The case report presented is unique due to the patient having an infection with *N. canis* as well as our patient having multiple true drug allergies to antibiotics that are used to treat Neisseria infections. The literature is sparse regarding infections from *N. canine* in humans.

Prior to 1982 there were no reported cases of *Neisseria canis* infections in humans, and very few have been reported since [6]. *N. canis* is considered normal flora in the oropharynx of dogs and cats, but can be an opportunistic pathogen in some cases [6,7]. The first case report of *N. canis* resulted from a cat bite, however there were no other case details mentioned [6]. A case report from 1989 of a cat bite wound culture resulted in the identification of *Pasteurella multocida, Eikenella corrodens, and N. canis*, with the *P. multocida *presumed as the primary pathogen and the infection was treated successfully with amoxicillin for one week [7]. A third case report in 1999 involved a purulent wound resulting from stepping on a dog bone. An isolate of *N. canis* was identified in the wound culture with susceptibility to benzylpenicillin, erythromycin, and tetracycline, but resistant to vancomycin [8]. Furthermore, another case report described a lung infection of *N. canis* that occurred in a patient with chronic obstructive pulmonary disease (COPD) with chronic bronchiectasis who was consistently exposed to the saliva of his dog. In this case, the *N. canis* was reported to be susceptible to ciprofloxacin, doxycycline, penicillin, amoxicillin-clavulanate, gentamicin, clindamycin, levofloxacin, erythromycin, and trimethoprim-sulfamethoxazole, however the patient was not treated with antibiotics due to mild symptoms [9]. 

According to the Infectious Diseases Society of America (IDSA), the preferred treatment for dog bites includes oral amoxicillin/clavulanic acid (Augmentin) or IV ampicillin/sulbactam (Unasyn) [10]. Alternative therapies could include second-generation cephalosporins, carbapenems, fluoroquinolones, or doxycycline all with anaerobic coverage of clindamycin or metronidazole [10]. In our patient, due to her multiple drug allergies, these treatment modalities could not be used. The patient in this case was treated with amoxicillin/clavulanic acid and metronidazole for seven days and resulted in a complete recovery. In many of the aforementioned cases, the patients were successfully treated with the preferred treatments for animal bites; however in this case, the treatment modality had to be modified due to our patient’s allergies.

## Conclusions

An infection from *N. canis* from an animal bite is rare and not well described in the literature. Although there exists a treatment modality for dog and cat bite infections, the case presented is unique due to our patient's multiple drug allergies and modification of treatment.
